# ARHGDIB Modulates Subcutaneous Fat Deposition in Ducks

**DOI:** 10.3390/ani16060975

**Published:** 2026-03-20

**Authors:** Mingyu Wang, Hao Zheng, Xing Chen, Ao Zhou

**Affiliations:** 1 Laboratory of Genetic Breeding, Reproduction and Precision Livestock Farming, School of Animal Science and Nutritional Engineering, Wuhan Polytechnic University, Wuhan 430023, China; 18736296503@139.com (M.W.); 15926744416@163.com (H.Z.); 2 Institute of Animal Husbandry and Veterinary, Wuhan Academy of Agricultural Science, Wuhan 430345, China

**Keywords:** duck, *ARHGDIB*, transcriptome sequencing, signaling pathways, adipogenesis

## Abstract

Background: Subcutaneous fat deposition critically impacts duck meat quality and feed efficiency. *ARHGDIB*, a Rho GTPase regulator implicated in adipogenesis, remains functionally uncharacterized in poultry. Methods: We monitored growth and fat deposition in ducks from 30 to 70 days, performed transcriptomics on adipose tissue, and established an in vitro duck preadipocyte model to assess *ARHGDIB* function via siRNA knockdown, Oil Red O staining, and RNA-seq. Results: Fat deposition peaked at 50 days. *ARHGDIB* expression was lowest in fat and decreased during differentiation. Its knockdown significantly enhanced lipid accumulation, upregulated *PPARγ* and *LPL*, and altered the expression of 1681 genes enriched in oxidative phosphorylation, insulin/TLR signaling, and autophagy pathways. Conclusions: *ARHGDIB* acts as a novel negative regulator of duck subcutaneous adipogenesis by suppressing differentiation and modulating an integrated metabolic-inflammatory network, offering a potential target for precision breeding.

## Simple Summary

Duck meat is a popular food, but too much fat beneath the skin can reduce its quality and make feeding less efficient. This study investigated a gene called ARHGDIB to understand its role in duck fat formation. We found that this gene is less active in fat tissue and its activity drops even further when fat cells are forming. By experimentally reducing the gene’s function in duck fat cells grown in the lab, we caused the cells to store more fat. This shows that *ARHGDIB* normally acts as a “brake” on fat formation. Understanding how this brake works could help breeders develop ducks with healthier levels of fat, leading to better meat quality for consumers.

## 1. Introduction

*Duck meat* is highly valued for its distinctive flavor and nutritional profile. However, excessive subcutaneous fat deposition, which accounts for approximately 70% of total body fat in *meat-type ducks*, detrimentally affects feed efficiency, carcass yield, and consumer acceptance [1,2]. Therefore, elucidating the molecular mechanisms governing subcutaneous adipogenesis is imperative for precision breeding strategies aimed at optimizing meat quality.

Adipocyte differentiation is orchestrated by a well-defined transcriptional cascade involving *PPARγ* and *C/EBPα*, which drive the expression of lipogenic enzymes such as *FAS* and *LPL* [3,4]. Despite this general framework, the specific regulators governing fat deposition in ducks, particularly within subcutaneous adipose tissue, remain largely unexplored. Recent genome-wide association studies have implicated *ARHGDIB* (Rho GDP dissociation inhibitor beta) in subcutaneous fat traits in Beijing ducks, highlighting its potential as a candidate regulator [5]. As a member of the RhoGDI family, *ARHGDIB* modulates Rho GTPase activity, cytoskeletal dynamics, and cell signaling—processes increasingly linked to adipogenesis and metabolic disorders [6,7]. However, its precise functional role and mechanistic contribution to adipocyte biology in avian species, or in any agricultural animal model, are completely unknown.

To address this critical knowledge gap, we conducted an integrated study combining in vivo growth analysis, transcriptomics, and in vitro functional assays in ducks. We hypothesized that *ARHGDIB* serves as a key regulator of subcutaneous preadipocyte differentiation. Our objectives were as follows: (1) characterize its expression pattern during duck growth and adipogenesis, (2) determine its functional impact on lipid accumulation through gain- and loss-of-function approaches, and (3) delineate the downstream transcriptional networks it governs. This work provides the first functional characterization of *ARHGDIB* in poultry adipogenesis, unveils its role as a novel negative regulator, and identifies associated signaling pathways, thereby offering fresh insights for targeted genetic improvement in duck breeding.

## 2. Materials and Methods

### 2.1. Growth Monitoring and Histological Analysis of Subcutaneous Adipose Tissue in Meat-Type Ducks

A total of 120 small-frame, high-quality *meat-type ducks* (Wuhan Institute of Animal Science and Veterinary Medicine) were raised to 70 d. The ducks were housed in floor pens with rice hulls as litter at a density of 3 birds/m^2^ under a natural light cycle, with ad libitum access to feed and water. The facility was naturally ventilated, and no artificial heating or cooling was applied beyond ambient conditions. Body weight was recorded at 10 d intervals from 30 d. At each interval, 10 ducks (sex-balanced) near the average weight underwent ultrasonic subcutaneous fat measurement. We acknowledge that selecting ducks near the average weight may introduce a selection bias, which we note as a limitation. After 12 h fasting, ducks were euthanized; subcutaneous adipose tissue was collected from 6 ducks for histology (4% paraformaldehyde fixation (Beijing Solarbio Science & Technology Co., Ltd., Beijing, China), paraffin embedding, 4 µm sectioning, H&E staining). Adipocyte cross-sectional area was quantified from ≥three 200× fields/section using Image-Pro Plus 6.0. All experimental procedures were approved by the Animal Care and Use Committee of Wuhan Polytechnic University (Approval No. WPU202311070). To ensure objectivity, the histological analysis was performed by a single investigator who was blinded to the sample age groups.

### 2.2. Tissue Sample Collection and RNA Extraction from Duck

Tissue samples (heart, liver, spleen, kidney, pectoral muscle, leg muscle, subcutaneous fat, abdominal fat, duodenum, thymus, and lung) were collected from three 28-day-old meat-type ducks after a 12 h fast and euthanasia. Tissues were snap-frozen in liquid nitrogen. Total RNA was extracted using TRIzol Universal (Vazyme Biotech Co., Ltd., Nanjing, China) reagent according to the manufacturer’s protocol. Briefly, tissues were homogenized with steel beads in TRIzol using a tissue grinder. After phase separation with chloroform, RNA was precipitated with isopropanol, washed with 75% ethanol, and dissolved in RNase-free water. RNA integrity was verified by agarose gel electrophoresis, and concentration/purity (A260/280 ratio 1.8–2.2) was measured using a Nanodrop One spectrophotometer (Thermo Fisher Scientific, Waltham, MA, USA).

### 2.3. Isolation and Culture of Subcutaneous Preadipocytes from Ducks

Subcutaneous preadipocytes were isolated from 2-week-old *WUQIN No.10 Meat-type Duck* (Both sexes were included) (Anas platyrhynchos domestica) (*n* = 6 ducks, 3 biological replicates) obtained from the Breeding Duck Farm of the Wuhan Institute of Animal Science and Veterinary Medicine. Preadipocyte identity was confirmed by their fibroblast-like morphology at confluence and their ability to differentiate and accumulate lipids upon induction with oleic acid, which was validated by Oil Red O staining. *Ducks* were euthanized via exsanguination through the carotid artery, and carcasses were disinfected in 1% Virkon S (Sigma-Aldrich, St. Louis, MO, USA) for 10 min. Subcutaneous adipose tissue was dissected and washed twice with pre-chilled PBS (Gibco, Grand Island, NY, USA) containing 2% penicillin–streptomycin (4 °C). Tissues were minced into 1 mm^3^ fragments, digested with 2% Type I collagenase (YEASEN, Shanghai, China) at 37 °C for 80 min (shaking every 5 min), and neutralized with equal-volume DMEM/F12 + 10% FBS (Gibco). Cells were pelleted (1000× *g*, 10 min), filtered through a 75 μm cell strainer (Corning, Corning, NY, USA), and cultured in DMEM/F12 + 10% FBS + 1% penicillin–streptomycin at 37 °C, 5% CO_2_. Media were changed every 2 days, and preadipocytes were used at passage 2–3. Three biological replicates were used for all in vitro experiments, defined as three independent cell isolations from three different ducks. Technical replicates refer to multiple wells from the same cell isolation.

### 2.4. Induction of Adipogenic Differentiation of Subcutaneous Preadipocytes

Preadipocytes were seeded into 12-well plates (1 × 10^5^ cells/well) and cultured to 100% confluence (Day 0). Induction medium (DMEM/F12 + 10% FBS + 1% penicillin–streptomycin + 300 μmol/L oleic acid (Solarbio, Beijing, China) was added, with medium replacement every 2 days. Differentiation was assessed on Day 4 via Oil Red O staining (endpoint: ≥80% cells with lipid droplets). Controls received vehicle (0.1% DMSO (Beijing Solarbio Science & Technology Co., Ltd., Beijing, China))-containing medium. Experiments included 3 biological replicates (*n* = 3 wells/replicate).

### 2.5. Oil Red O Staining

Oil Red O staining was performed using an Oil Red O staining kit (Solarbio, Beijing, China). Initially, the cell culture medium was removed, and the cells were washed twice with PBS buffer. Subsequently, 500 μL of Oil Red O fixative was added to each well, and the cells were fixed at room temperature for 25 min. After fixation, the fixative was discarded, and the cells were washed twice again with PBS buffer. Next, 500 μL of 60% isopropanol was added to each well for a 30 s rinse. The isopropanol was then discarded, and freshly prepared Oil Red O staining solution (300 μL of staining solution A mixed with 200 μL of staining solution B, allowed to stand for 10 min, and then filtered through a 0.22 μm filter) was added. The cells were stained for 20 min in the dark. After staining, the staining solution was removed, and the cells were rinsed with 500 μL of 60% isopropanol for 30 s until the interstitial parts of the cells were clearly visible. Finally, the cells were washed with PBS buffer until no residual staining solution remained. After washing, 500 μL of Oil Red O buffer was added to cover the cells, and the cells were observed and photographed under a microscope to assess the degree of adipocyte differentiation.

### 2.6. Transfection

Preadipocytes (50% confluence) were transfected with ARHGDIB-specific siRNA (sequence: 5′-CAUUCAUGGUUGGCAGCUATT-3′; Jima, Guangzhou, China) or scrambled siRNA (control) using jetPRIME (Polyplus, Illkirch, France) at 30 nM. Transfection efficiency was verified by RT-qPCR (≥70% knockdown) and Western blot.

### 2.7. Total RNA Extraction and cDNA Synthesis

Total RNA was extracted using TRNzol Universal (TIANGEN, Beijing, China). RNA integrity was assessed via 1% agarose gel electrophoresis (RIN ≥ 8.0, Agilent 2100 Bioanalyzer (Agilent Technologies, Santa Clara, CA, USA)). DNase I (Thermo Fisher (Waltham, MA, USA)) treatment was performed to remove genomic DNA. cDNA was synthesized using HiScript III RT SuperMix (+gDNA wiper) (Vazyme, Nanjing, China) and stored at −20 °C.

### 2.8. RT-qPCR

Quantitative PCR primers were designed, and real-time PCR was performed using the Taq Pro Universal SYBR qPCR Master Mix (Vazyme, China). cDNA was used as the template, with the duck β-actin gene serving as the internal control. The reaction mixture consisted of the following components: 5 μL SYBR Green, 1 μL cDNA, 3.6 μL nuclease-free water, and 0.2 μL of each forward and reverse primer. RT-qPCR was subsequently carried out under the appropriate thermal cycling conditions. The sequences of the quantitative primers are provided in Table 1.

### 2.9. RNA-Seq

RNA-seq Libraries were constructed using the Illumina NEBNext Ultra II RNA Library Prep Kit, with insert sizes of 200–300 bp (Agilent 2100 Bioanalyzer). Sequencing was performed on an Illumina NovaSeq 6000 platform (2 × 150 bp, 10 Gb/library). Raw data were filtered (Trimmomatic v0.39) to remove adapters and low-quality reads (Q < 20). Clean reads were aligned to the duck reference genome (ASM874695v1, Ensembl) using HISAT2 v2.2.1 (mapping rate ≥ 90%). Differential expression was analyzed via DESeq2 v1.38.3 (adjusted *p*-value (*padj*) < 0.05). GO/KEGG enrichment used clusterProfiler v4.8.1. PPI analysis was performed via STRING v11.5 (confidence score ≥ 0.700).

### 2.10. Western Blot

Proteins were extracted using RIPA lysis buffer (Beyotime, Shanghai, China) with protease inhibitors. Concentration was measured via BCA assay (Vazyme, Nanjing, China). Samples (20 μg/lane) were separated by 12% SDS-PAGE and transferred to PVDF membranes (Millipore, Burlington, MA, USA). Membranes were blocked with 5% skim milk (1 h, room temperature) and incubated with anti-*ARHGDIB* (1:1000, Abcam, Cambridge, UK; FNab00555) or anti-β-actin (1:5000, Proteintech, Rosemont, IL, USA; GB11001) overnight at 4 °C. Secondary antibodies (HRP-conjugated goat anti-rabbit IgG, 1:5000, Proteintech) were incubated for 1 h. Signals were detected via ECL (Vazyme, Nanjing, China) and imaged (ChemiDoc XRS+ (version 5.1), Bio-Rad (Hercules, CA, USA)).

### 2.11. Statistical Analysis

Data are presented as mean ± SD (*n* = 3 biological replicates, with 3 technical replicates per biological replicate for qPCR and Oil Red O quantification). Data distribution was tested for normality using the Shapiro–Wilk test, and homogeneity of variances was assessed by Levene’s test. Student’s *t*-test (two groups) or one-way ANOVA (multiple groups) was used, with *p* < 0.05 considered significant. For one-way ANOVA, when significant differences were detected, Tukey’s post hoc test was used for multiple comparisons. GraphPad Prism 9.5 was used for visualization.

## 3. Results

### 3.1. Growth and Adipose Development in Meat Ducks (30–70 Days)

**Figure 1:**
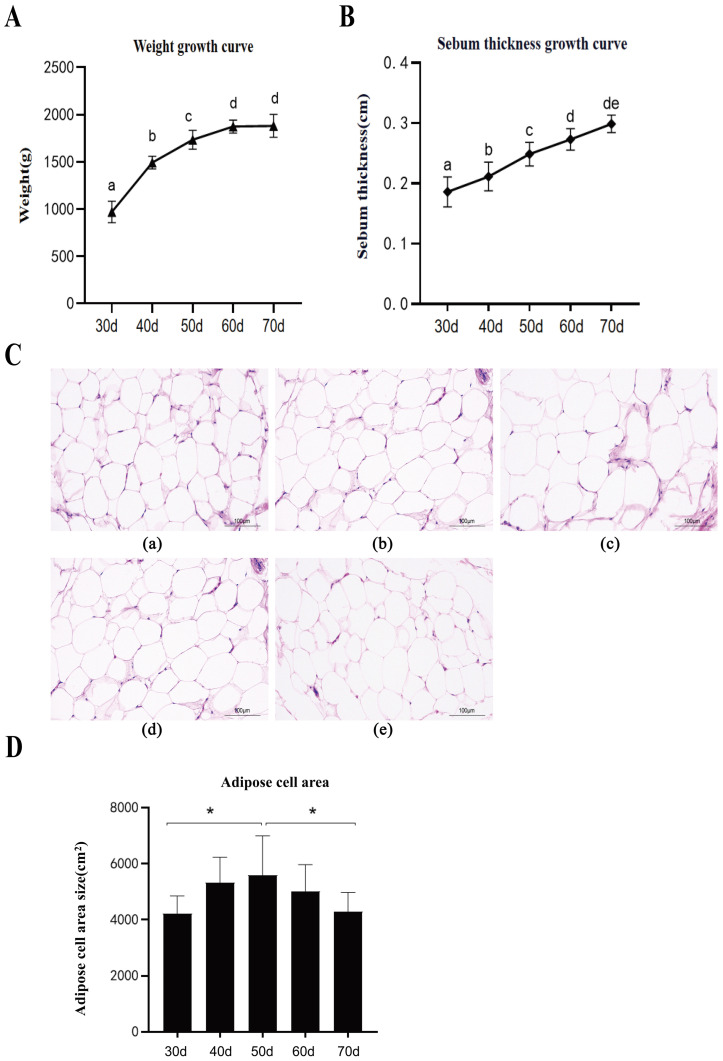
Growth performance and subcutaneous adipose tissue development in meat-type ducks from 30 to 70 d of age. (**A**,**B**) (**A**) Body weight and (**B**) subcutaneous fat thickness at different ages. Different lowercase letters indicate significant differences among ages at *p* < 0.01, and different uppercase letters indicate significant differences at *p <* 0.05. Ages with the same letter are not significantly different (*p* > 0.05). (**C**) Representative hematoxylin and eosin (H&E)-stained sections of subcutaneous adipose tissue at (**a**) 30, (**b**) 40, (**c**) 50, (**d**) 60, and (**e**) 70 d of age. Scale bars = 100 µm. (**D**) Quantitative analysis of subcutaneous adipocyte cross-sectional area. Data are presented as mean ± SEM (*n* = 6). Asterisks indicate significant differences compared with the 30 d group (*p <* 0.05).

A total of 120 meat-type ducks were reared to 70 d under standard conditions. Body weight and ultrasonic subcutaneous fat thickness were recorded at 10 d intervals from 30 d of age. Both parameters increased significantly (*p* < 0.05) from 30 to 70 d, with a faster growth rate (*p* < 0.01) observed between 30 and 50 d (Figure 1A,B). Histological analysis of subcutaneous adipose tissue (30–70 d) revealed peripheral localization of adipocyte nuclei and a reduction in adipocyte number (Figure 1C). Quantitatively, adipocyte area increased (*p* < 0.05) from 30 to 50 d but decreased (*p* < 0.05) thereafter (Figure 1D).

### 3.2. Key Lipid Metabolism Genes in Duck Fat: Transcriptomics and Tissue Profiles

**Figure 2:**
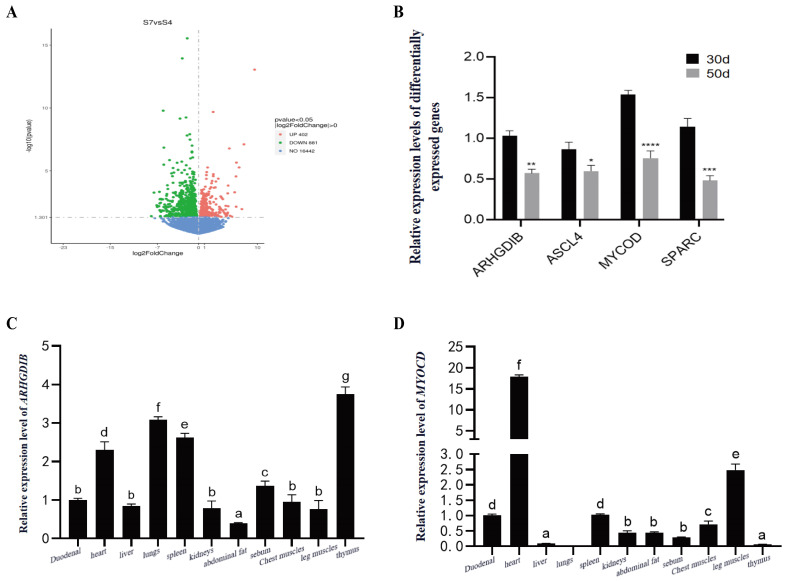
Transcriptomic analysis of subcutaneous adipose tissue between 30 and 50 d of age in meat-type ducks. (**A**) Volcano plot displaying differentially expressed genes (DEGs) between 30 and 50 d of age. Significantly upregulated genes are shown in red, and significantly downregulated genes are shown in green. The thresholds for significance were set at |log_2_ fold change| > 1 and adjusted *p* < 0.05. (**B**) Validation of selected DEGs by quantitative real-time PCR. Data are presented as mean ± SEM (*n* = 6). Asterisks indicate significant differences between 30 and 50 d of age (*p* < 0.05; * *p* < 0.05, ** *p* < 0.01, *** *p* < 0.001, **** *p* < 0.0001). (**C**,**D**) Tissue expression profiles of ARHGDIB (**C**) and MYOCD (**D**). Different lowercase letters above the bars indicate significant differences among tissues at *p* < 0.05. Tissues sharing a common letter are not significantly different.

Transcriptome sequencing was conducted on subcutaneous adipose tissue collected at 30 and 50 days of age. Using thresholds of |log_2_fold change| > 1 and a *p*-value < 0.05, 1283 differentially expressed genes (DEGs) were identified, comprising 881 downregulated and 402 upregulated genes (Figure 2A). Four DEGs related to lipid metabolism were selected for validation by quantitative real-time PCR (qPCR) using the 2^^−ΔΔCt^ method, and their expression trends were consistent with the sequencing data (Figure 2B). Furthermore, tissue expression profiles of *ARHGDIB* and *MYOCD* were analyzed (Figure 2C,D). *ARHGDIB* was widely expressed in all examined tissues, with the highest expression observed in the thymus and the lowest in subcutaneous fat. Although expressed across multiple tissues, the distribution pattern—characterized by its minimal level in subcutaneous fat—suggests that *ARHGDIB* may have a high baseline expression required for the development of immune-related tissues or for involvement in immune regulatory pathways. The relatively low expression level in adipose tissue implies that local regulation or a transient expression peak of *ARHGDIB* during subcutaneous adipogenesis may play a critical role in fat deposition. Further research is necessary to elucidate the transcriptional and epigenetic regulatory mechanisms underlying this tissue-specific expression pattern. *MYOCD* expression was most abundant in the heart, followed by leg muscle.

### 3.3. Expression Dynamics of ARHGDIB During Adipocyte Differentiation

**Figure 3:**
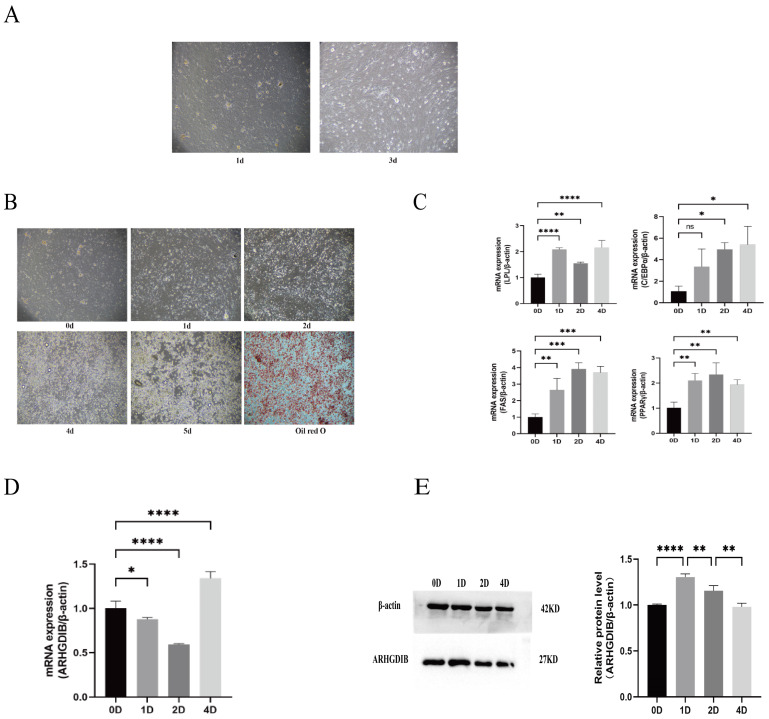
Adipogenic differentiation of duck subcutaneous preadipocytes and *ARHGDIB* expression dynamics. (**A**) Phase-contrast microscopy images at 24 and 72 h post-seeding. Scale bar = 100 µm. (**B**) Quantitative Oil Red O staining of lipid accumulation on Days 0, 1, 2, 4 and 5 post-induction. Values are mean ± SD; * *p* < 0.01 vs. Day 0 (one-way ANOVA). (**C**) Relative mRNA expression of adipogenic markers (*LPL*, *C/EBPα*, *PPARγ*, *FAS*) during early differentiation (Days 1–2). Values are mean ± SD (*n* = 3 biological replicates); *p* < 0.05 vs. Day 0 (Student’s *t*-test). (**D**,**E**) ARHGDIB mRNA (**D**) and protein (**E**) levels during differentiation; β-Actin as loading control. Values are mean ± SD (*n* = 3 biological replicates); * *p* < 0.05, ** *p* < 0.01, *** *p* < 0.001, **** *p* < 0.0001 vs. Day 0. Experiments were performed with *n* = 3 biological replicates.

Primary subcutaneous preadipocytes from 1- to 2-week-old *WUQIN No.10 Meat-type Ducks* (*n* = 6 ducks, 3 biological replicates) reached >90% confluence within 72 h (Figure 3A). Oleic acid induction for 4 days triggered differentiation, confirmed by quantitative Oil Red O staining: lipid droplets were extracted with 100% isopropanol and *OD510* measured (three technical replicates per sample; *p* < 0.01 vs. Day 0, Figure 3B). Temporal expression of adipogenic markers *LPL*, *C/EBPα*, *PPARγ*, and *FAS* was analyzed. In the subcutaneous adipogenesis model used in this study, *FAS* expression did not exhibit significant time-dependent changes. This result suggests that the driving role of *FAS* in early subcutaneous adipogenesis may be less direct than that of genes such as *LPL*, or that its expression is subject to regional regulatory influences. Further studies should incorporate the spatiotemporal dynamics of fatty acid supply and lipid transport to better elucidate its specific role in this process (Figure 3C). ARHGDIB mRNA and protein declined significantly by Day 2, returning to basal levels by Day 4 (Figure 3D,E), indicating stage-specific suppression during differentiation.

### 3.4. Functional Validation of ARHGDIB in Adipogenesis

**Figure 4:**
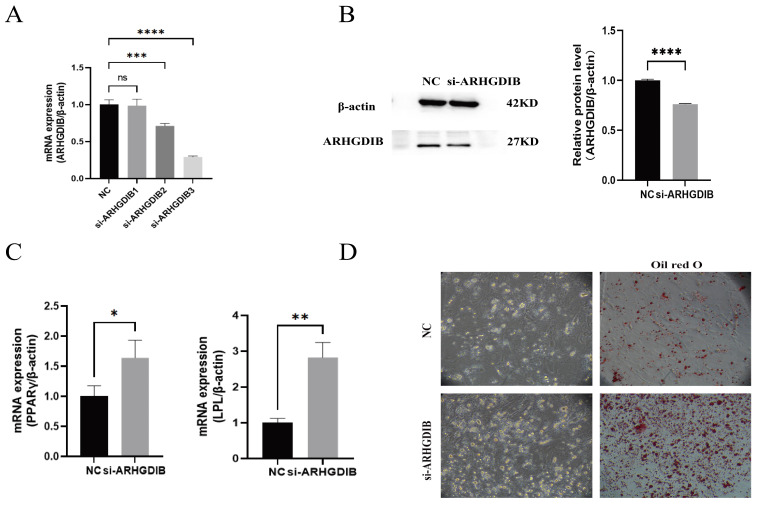
Functional validation of *ARHGDIB* knockdown in duck preadipocytes. (**A**) mRNA knockdown efficiency of three *ARHGDIB*-specific siRNAs. (**B**) Protein knockdown efficiency assessed by Western blot; β-actin as loading control. (**C**) Relative mRNA levels of *PPARγ* and *LPL* after si-RNA3 transfection. (**D**) Quantitative Oil Red O staining showing lipid droplet formation. Scale bar = 100 µm. Lipids extracted and OD_510_ measured (three technical replicates). Values are mean ± SD (*n* = 3 biological replicates); * *p* < 0.05, ** *p* < 0.01, *** *p* < 0.001, **** *p* < 0.0001 vs. NC (one-way ANOVA). All experiments used *n* = 3 biological replicates and three technical replicates for qPCR/Western blot.

Three siRNA sequences targeting *ARHGDIB* were designed; siRNA3 yielded maximal knockdown (≥80% mRNA reduction, *p* < 0.01; ≥70% protein reduction, Figure 4A,B) and was used for subsequent tests. Knockdown significantly increased mRNA levels of *PPARγ* and *LPL* (Figure 4C) and elevated lipid droplet numbers (quantified by *OD510*; *p* < 0.01 vs. NC, Figure 4D). These results demonstrate that *ARHGDIB* suppresses duck preadipocyte differentiation and may negatively regulate subcutaneous fat deposition.

### 3.5. Transcriptome Sequencing

**Figure 5:**
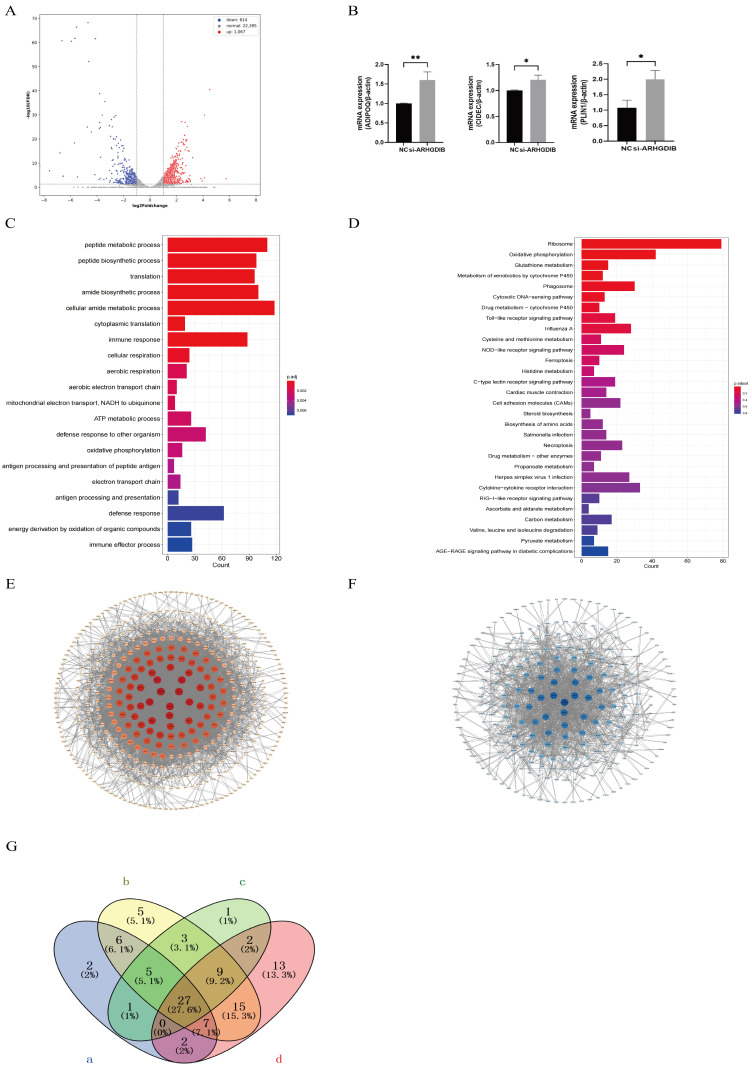
Transcriptomic analysis of ARHGDIB knockdown effects. (**A**) Volcano plot of differentially expressed genes (DEGs) between si-ARHGDIB and NC groups (*padj* < 0.05). (**B**) RT-qPCR validation of ADIPOQ, CIDEC, and PLIN1. Values are mean ± SD (*n* = 3 biological replicates); *p* < 0.05 vs. NC. (**C**) Top enriched GO biological processes for DEGs. (**D**) Top enriched KEGG pathways for DEGs. (**E**,**F**) Protein–protein interaction (PPI) network of DEGs (STRING confidence ≥ 0.700, visualized in Cytoscape v3.10.2). Node size and color indicate degree of connectivity. (**G**) Venn diagram of 27 shared DEGs among autophagy, oxidative phosphorylation, insulin signaling, and Toll-like receptor pathways. RNA-seq data based on *n* = 3 biological replicates per group; RT-qPCR performed with three technical replicates. * *p* < 0.05, ** *p* < 0.01. Purple represents the autophagy pathway, yellow represents the oxidative phosphorylation pathway, red represents the insulin signaling pathway, and green represents the Toll-like receptor pathway.

*ARHGDIB*-siRNA-transfected cells (3 biological replicates) were sampled 48 h post-induction. RNA-seq (Illumina NovaSeq 6000, 10 Gb/library) identified 1681 DEGs (1067 up, 614 down; adjusted *p*-value (*padj*) < 0.05, Figure 5A). RT-qPCR validation of *ADIPOQ*, *CIDEC*, *PLIN1* showed consistent upregulation in *si-ARHGDIB* group (*p* < 0.05, Figure 5B). GO analysis highlighted enrichment in peptide metabolism, translation, immune response, oxidative phosphorylation, and defense response (Figure 5C). KEGG pathways included Ribosome, oxidative phosphorylation, glutamate metabolism, P450 xenobiotic metabolism, Toll-like receptor signaling, Avian influenza, and cytokine–receptor interaction (Figure 5D). PPI network (STRING, confidence ≥ 0.700; Cytoscape 3.10.2) visualized interaction degrees (Figure 5E,F). Venn analysis of autophagy, oxidative phosphorylation, insulin signaling, and TLR pathways revealed 27 shared DEGs (Figure 5G), implicating *ARHGDIB* in regulating these key genes for subcutaneous fat deposition.

## 4. Discussion

Subcutaneous fat deposition critically influences feed efficiency and meat quality in duck production. Consistent with previous findings [8,9], we observed a significant increase in both body weight and subcutaneous fat thickness from 30 to 70 days of age. Histological analysis further revealed that adipocyte maturation was complete by 30 days, with cell area peaking around 50 days, indicating an active phase of fat deposition during this period.

Through comparative transcriptomic analysis of adipose tissue at 30 and 50 days, we identified *ARHGDIB* as a potential regulator of this process. Notably, *ARHGDIB* expression was significantly lower in subcutaneous fat compared to various other tissues, suggesting a tissue-specific regulatory role. *ARHGDIB* encodes a Rho GDP dissociation inhibitor known to modulate cytoskeletal dynamics by stabilizing inactive Rho GTPases [10]. During the in vitro differentiation of duck preadipocytes, both mRNA and protein levels of *ARHGDIB* declined. Functional knockdown of *ARHGDIB* significantly enhanced lipid accumulation and upregulated key adipogenic markers such as *PPARγ* and *LPL*, establishing its role as a negative regulator of adipogenesis in ducks. *FAS* did not show significant changes in this study, suggesting that its role in the early differentiation of subcutaneous adipocytes may be relatively limited. Future research should integrate the dynamics of fatty acid supply, lipid transport, and cross-tissue coordination with hepatic and muscle metabolism to further elucidate the specific mechanisms of *FAS* in duck subcutaneous fat deposition.

To elucidate the underlying mechanisms, we performed transcriptome sequencing following *ARHGDIB* knockdown, which identified 1681 differentially expressed genes (DEGs). Enrichment analysis highlighted several key pathways: oxidative phosphorylation, insulin signaling, Toll-like receptor (TLR) signaling, and autophagy. The activation of insulin signaling likely promotes glucose uptake and lipogenesis [11], while upregulated oxidative phosphorylation supplies the necessary ATP for lipid synthesis [12]. Alterations in TLR signaling may modify the inflammatory microenvironment, indirectly supporting adipogenesis [13], and autophagy may regulate lipid turnover through lipophagy [14]. The coordinated enrichment of these pathways—supported by 27 overlapping genes—suggests that *ARHGDIB* may act as a regulator of an integrated network governing energy metabolism and inflammatory responses during fat deposition. However, this network hypothesis requires direct functional validation. Future work should directly measure pathway activities, such as the phosphorylation status of insulin signaling components, mitochondrial respiration rates, ATP production, and levels of inflammatory cytokines, to validate the regulatory role of *ARHGDIB* suggested by our transcriptomic findings.

Notably, this network perspective is further reinforced by several validated DEGs with established functional links to adipogenesis and lipid metabolism. Specifically, *CMKLR1* promotes adipocyte differentiation and energy balance [15,16]; methylation of *SLC22A3* is associated with hyperglycemia and obesity phenotypes [17,18]; *RSAD2* modulates lipid biosynthesis and lipid droplet content [19,20]; *TRIM25* acts as an E3 ubiquitin ligase targeting *PPARγ*, thereby limiting adipogenesis [21]; and *BLVRA* influences insulin signaling and white adipose tissue remodeling [22,23]. Based on these integrated findings, we propose that *ARHGDIB* restrains subcutaneous fat deposition in ducks by simultaneously inhibiting adipocyte differentiation and suppressing a pro-adipogenic transcriptional program that coordinates metabolic and immune pathways.

Nevertheless, certain limitations must be acknowledged. First, all functional experiments were performed in vitro using primary cell cultures; therefore, the role of *ARHGDIB* in vivo remains to be confirmed through animal models, such as gene overexpression or knockdown studies in ducks via viral vectors or the establishment of transgenic lines. Second, while three biological replicates for the RNA-seq experiment are common and yielded robust data, a larger sample size would provide even greater statistical power. Third, our study focuses on transcriptional changes; we did not perform direct functional assays of metabolic pathway activity (e.g., measurement of oxygen consumption rate, ATP production, or inflammatory cytokine secretion) or metabolite profiling, which are essential to confirm the inferred pathway activities. Fourth, the upstream factors—such as specific transcription factors or epigenetic modifications (e.g., DNA methylation or histone acetylation)—that govern the downregulation of *ARHGDIB* during the early stages of differentiation remain unknown and warrant investigation. Future studies should address these gaps by employing rescue experiments, pathway-specific inhibitors, and in vivo models to establish direct causality, elucidate the regulatory mechanisms controlling *ARHGDIB* expression, and assess the translational potential of targeting *ARHGDIB* for improving duck meat quality.

## 5. Conclusions

This study provides the first functional evidence that *ARHGDIB* acts as a negative regulator of subcutaneous adipogenesis in ducks. Knockdown of *ARHGDIB* in vitro promotes lipid accumulation and alters the expression of genes involved in key metabolic and inflammatory pathways, as revealed by transcriptomic analysis. These findings suggest that *ARHGDIB* may regulate adipocyte differentiation through mechanisms involving Rho GTPase signaling. While this work offers fundamental insights into the molecular basis of fat deposition in poultry, the precise mechanisms by which *ARHGDIB* coordinates these pathways, as well as its potential as a target for genetic improvement in duck breeding, require further validation through in vivo studies and direct functional assays.

## Figures and Tables

**Table 1:** RT-qPCR Primer sequences.

Gene Name	Forward Primer (5′-3′)	Reverse Primer (5′-3′)
β-actin	ATGTCGCCCTGGATTTCG	CACAGGACTCCATACCCAAGAA
ARHGDIB	CTGAAATACGTGCAGCATACCTACC	GTGAACTCATTCTGTCCATTCCTTC
ACSL4	CAAACCTGGAAGCCCCTACCGT	CAGTCAGTCCACTCCCCAAGCG
MYOCD	AAGAGCCCGCAGCACATCA	TCAATCAGCACGTCCAGGAGTT
SPARC	TCCTGCCATTTCTTCGCCA	ACGTTCTTCAGCCAGTCCCG
LPL	ACAATGTCCACTTGCTGG	TAGGTGTGTAGGACATCC
C/EBPα	GTGCTTCATGGAGCAAGCCAA	TGTCGATGGAGTGCTCGTTCT
PPARγ	TTCCAACTCCCTTATGGC	GGCATTGTGTGACATTCC
FAS	TCAACCTTCTGCTGAAGC	ACTTCTGCACCTGCTTCAG
ADIPOQ	CAAGGTCAGCCTCTACAAGAAGGA	AGCCCATGAAAGTGGAATCGT
PLIN1	TGGAAGTGCCAAGGAGAACG	CTCGTAGACCTCACACACGG
CIDEC	CTACCAGCTGAGCCTTTCCC	TAGAGCGTGGCTTTGACGTT

## Data Availability

The raw sequence data reported in this paper have been deposited in the OMIX database, China National Center for Bioinformation/Beijing Institute of Genomics, Chinese Academy of Sciences (https://ngdc.cncb.ac.cn/omix) (accessed on 17 March 2026). The dataset can be accessed via the accession number OMIX015506 with the following preview link: https://ngdc.cncb.ac.cn/omix/preview/nL9TWxcz (accessed on 17 March 2026). All other relevant data supporting the findings of this study are available within the article.
